# Lysosomal iron modulates NMDA receptor-mediated excitation via small GTPase, Dexras1

**DOI:** 10.1186/s13041-016-0220-8

**Published:** 2016-04-14

**Authors:** Rachel S. White, Anup K. Bhattacharya, Yong Chen, Madeleine Byrd, Mary F. McMullen, Steven J. Siegel, Gregory C. Carlson, Sangwon F. Kim

**Affiliations:** Department of Psychiatry, Center for Neurobiology and Behavior, Perelman School of Medicine at the University of Pennsylvania, Philadelphia, PA 19104 USA; Department of Systems Pharmacology and Translational Therapeutics, Perelman School of Medicine at the University of Pennsylvania, 125 S 31st, TRL RM 2207, Philadelphia, PA 19104 USA

## Abstract

**Background:**

Activation of NMDA receptors can induce iron movement into neurons by the small GTPase Dexras1 via the divalent metal transporter 1 (DMT1). This pathway under pathological conditions such as NMDA excitotoxicity contributes to metal-catalyzed reactive oxygen species (ROS) generation and neuronal cell death, and yet its physiological role is not well understood.

**Results:**

We found that genetic and pharmacological ablation of this neuronal iron pathway in the mice increased glutamatergic transmission. Voltage sensitive dye imaging of hippocampal slices and whole-cell patch clamping of synaptic currents, indicated that the increase in excitability was due to synaptic modification of NMDA receptor activity via modulation of the PKC/Src/NR2A pathway. Moreover, we identified that lysosomal iron serves as a main source for intracellular iron signaling modulating glutamatergic excitability.

**Conclusions:**

Our data indicates that intracellular iron is dynamically regulated in the neurons and robustly modulate synaptic excitability under physiological condition. Since NMDA receptors play a central role in synaptic neurophysiology, plasticity, neuronal homeostasis, neurodevelopment as well as in the neurobiology of many diseases, endogenous iron is therefore likely to have functional relevance to each of these areas.

## Background

Although bio-available iron is essential for normal neurological function, iron deficiency or excess iron can produce serious neurological consequences. Examples of CNS iron misregulation associated with diseases include Friedreich’s ataxia, associated with neuronal and myocardial mitochondrial iron accumulation [1, 2], Hallervorden-Spatz syndrome (defect on pantothenate kinase 2 - PANK2), characterized by marked iron overload in the globus pallidus [3], aceruloplasminemia, generated by an iron overload in basal ganglia [4], and Parkinson’s disease, where iron accumulation is found in the substantia nigra [5]. Iron deficiency both prenatally and perinatally have also been associated with intellectual disabilities and psychiatric disorders [6–9]. In each of these diseases, iron deregulation is associated with the pathophysiology, yet it has been difficult to assess a physiologic role played by iron in the brain. However, lack of iron leads to impairment in hippocampal electrophysiology, CA1 apical dendrite structure abnormality and deficits in learning and memory, indicating a physiologic role for iron is present [10, 11].

To investigate iron modulation of neuronal physiology, we focused on synaptic transmission and in particularly the role of N-methyl-D-aspartate receptors (NMDA-Rs). These are glutamate-gated ion channels widely expressed in the central nervous system that play key roles at glutamatergic transmission both within synapses and extrasynaptically. Our prior data, as well as recent work by others, demonstrate that extracellular iron can impact NMDA-R dependent neurotoxicity [10, 12, 13]. We showed that glutamate/NMDA neurotransmission modulates neuronal iron trafficking via a small GTPase, Dexras1 [13, 14]. Following activation of neuronal Nitric Oxide Synthase (nNOS), *S*-nitrosylation of Dexras1 by NO induces iron transport via the Divalent Metal Transporter (DMT1) [13–15]. Thus we know that Dexras1 in complex with DMT1 can transport extracellular iron across the cell membrane [16, 17]. There are two isoforms of DMT1, which are localized indifferent cellular compartments due to differences in 20 amino acids at the C-terminus. Localization studies revealed that isoform II has higher cell surface expression and is efficiently sorted to recycling endosomes upon internalization. By contrast, isoform I is targeted to lysosomes [18]. A voltage clamp study showed that each isoform has the same functional efficiency as an iron transporter [19]. Thus, while prior work has focused on the impact of extracellular iron, these studies targeted a potential intracellular role for DMT1 that may be better suited to modulate normal phUsing iron imaging in brain slices, we first show that iron is available for signaling in the hippocampus. Next, combining imaging and electrophysiology we demonstrate that this intracellular iron is sensitive to activity and that iron specifically modulates NMDA-Rs to impact synaptic excitability. Finally, we find that the Dexras1/DMT1 pathway in lysosome is necessary to generate intracellular iron signaling, and that disruption of the intact lysosomal function impacts iron signaling. Together our findings identify a novel iron signaling pathway with robust affects on NMDA-R dependent excitability.

## Results

### Iron is released into the hippocampal cytosol in an activity dependent manner

**Fig. 1:**
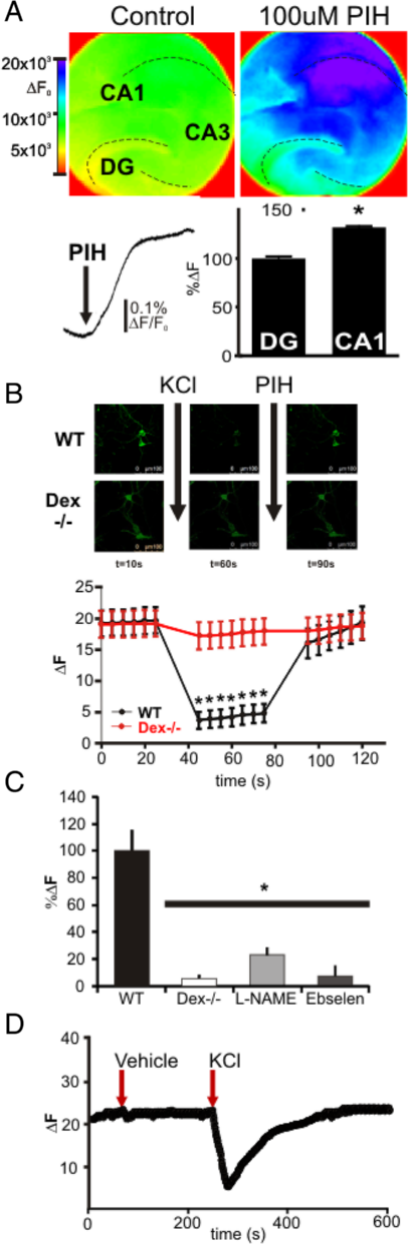
Liable iron is available in CA1 and is released in an activity dependent manner. **a**. Following application of the iron sensitive dye Calcein-AM, CA1 had the largest response to PIH application when compared to DG and CA1 (*n* = 5). Top 2 panels show pseudocolor images of hippocampal slices dyed with Calcein-AM dye both before (left) and after 100 μM PIH (Right; an average of 15 s). Purple indicates the biggest change in fluorescence. Bottom left shows the change in fluorescence over time normalized to baseline fluorescence, and the bar graph is an average of the change in fluorescence in CA1 and DG (*p < 0.05). **b.** Live cell imaging was performed with primary hippocampal neurons using fluorescence-based iron de-quenching imaging method with Calcein-AM dye (*n* = 7). Depolarization of the neurons with 50 mM KCl induces a 77 % decrease in fluorescence. When PIH is added to the culture, fluorescence returns to 89 % of control (*p* < 0.001). In contrast there is no decrease in fluorescence in Dexras1 KO hippocampal cultures when PIH is applied. **c**. Summary of live cell imaging with inhibitors which block nNOS and DMT1. Primary hippocampal neurons are pretreated with inhibitors for 30 min and an imaging experiment was performed as shown in Fig B. Inhibitor treatment significantly reduced the iron released into the cytosol (*p* < 0.05). **d**. Approximately 2,000 primary hippocampal neurons were seeded in 96 well plates and labeled with PhennGreen SK dye (20 μM). The fluorescent signal was measured by BioTek multiplate reader linked with automated reagent dispenser for KCl (a final concentration of 50 mM) (*n* = 3). The y-axis is arbitrary fluorescence change

We have previously shown that neurotransmitter dynamically modulates trafficking of extracellular iron via the plasma membrane in neurons leading to the excitotoxicity [14]. What is less known is whether or how cytosolic iron might affect the homeostasis of the excitability of neurons. To investigate whether there is detectable intracellular iron for signaling in the cytosol of neurons we stained mouse hippocampal brain slices with the heavy metal sensitive dye calcein-AM. Calcein-AM passes through the plasma membrane and reacts with cytosolic unspecific esterases producing calcein, which is retained within the cytosol of cells. Calcein fluorescence is quenched following chelation of low-mass labile iron, as well as other polyvalent metals therefore it binds iron the most effectively at cytosolic pH (despite its name it effectively binds to calcium or magnesium only at alkaline pH) [20, 21]. To identify if there was cytosolic iron in normal conditions, we measured calcein de-quenching, or an increase in fluorescence, following treatment of slices by a membrane permeable iron chelator, 100 μM pyridoxal isonicotinoyl hydrazine (PIH) [22–24] as combination of Calcein dye with an iron chelator will provide iron specific fluorescent signal. Following PIH application there was an increase in fluorescence in the CA1 region of the hippocampus (32 ± 6 %, *p* < 0.001, *n* = 4 slices, Fig. 1a). This effect was only seen with a membrane permeable iron chelator as neither non-permeable iron chelator (deferioxamine) nor other divalent metal chelators such as tetrathiomolybdate produce de-quenching of calcein-AM (data not shown). Activation of dissociated hippocampal neurons by 50 mM KCl also quenched the calcein-AM signal while there is no change in fluorescent signal in neurons from Dexras1 KO mice suggesting an activity dependent release of iron into the cytoplasm of the cell via Dexras1 (−77 ± 1.6 %; *p* < 0.0001, *n* = 7) (Fig. 1b). Again supporting activity dependent iron release the fluorescent signal was de-quenched by addition of membrane permeable iron specific, chelator, PIH (89 ± 3.5 %, *p* < 0.0001, *n* = 7). In a previous study we have shown that Dexras1-mediated iron flux is modulated by nNOS as well as DMT1, an iron channel [16, 17]. Therefore we pharmacologically blocked these enzymes utilizing L-NAME (1 mM) or ebselen (10 uM) respectively and found that an inhibition of either nNOS or DMT1 blocked iron release into cytosol (Fig. 1c). To further validate these results, we measured a change in fluorescent signal utilizing an alternative iron-sensitive dye, PhenGreen-SK, which is composed of phenanthroline coupled to Flourescein [25, 26]. This dye like calcein-AM is insensitive to major intracellular ions including endogenous levels of calcium. In this experiment, we detected a signal with a 96 well fluorescence microplate reader and observed that the signal was rapidly quenched upon neuronal cell depolarization suggesting a rapid release of iron into cytosol (Fig. 1d).

### Decreasing cytosolic iron pool enhances synaptic excitability in the hippocampus

**Fig. 2:**
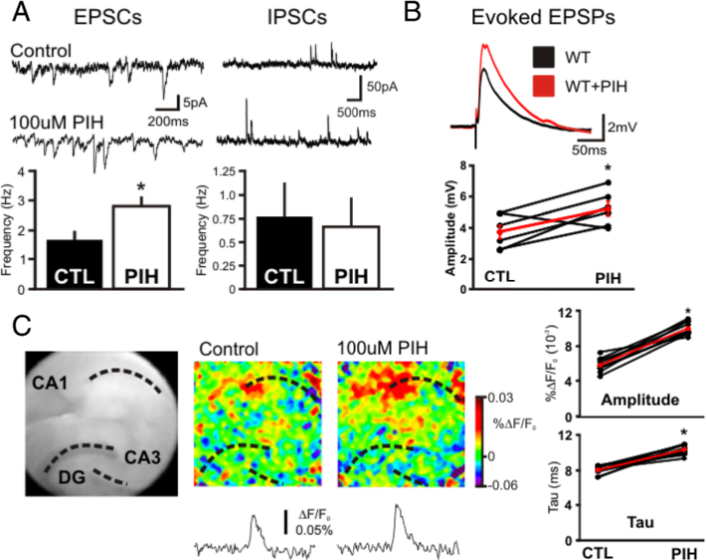
Chelation of iron modifies the excitability of pyramidal cells and circuit of the hippocampus. **a**. Whole cell patch clamp of CA1 pyramidal cells shows that 100 μM PIH application induces an increase in spontaneous EPSC frequency from 1.7 ± 0.28Hz to 2.8 ± 0.35Hz (*p < 0.05). On the right, there is no change in IPSC with PIH. Top shows representative traces from an experiment and bottom are bar graphs of cumulative data. **b**. There is an increase in the evoked EPSP in PIH. On the top is example recordings of whole cell current clamp and bottom is a graph showing population (black) and average data (red) in the WT animals both before and after 100 μM PIH application. PIH causes an increase in the amplitude of the evoked EPSP in CA1 pyramidal cells (Control: 3.7 ± 0.4 mV, PIH: 5.23 ± 0.4 mV, *n* = 6, *p* < 0.05). **c.** Shows grey scale image of a hippocampal slice (on the left) and snapshots of peak responses to stimulation in control (middle) and 100 μM PIH (right) conditions (average of 30 ms). Underneath are the traces of an average response of pixel fluorescence signals over time from a standardized region of CA1 in both conditions. There is an enhancement of the response following PIH application. Population line graphs of the VSD experiments as well as the average (red) confirm that there is a consistent increase in both amplitude of EPSP and time to decay back to baseline (tau) (*n* = 10, *p < 0.05)

**Fig. 3:**
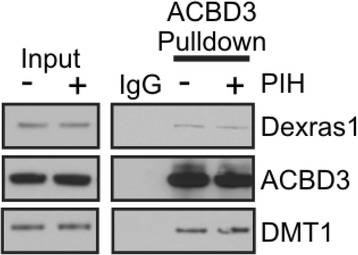
Iron status does not affect a formation of ternary complex among Dexras1/ACBD3/DMT1**.** Brain slices were treated with PIH as described in Figs. 2 and 3. A treatment of tissues with PIH, iron chelator did not change interaction between Dexras1, ACBD3 and DMT1

To investigate if the chelatable iron pool affects neuronal excitability we studied the impact of iron chelation on synaptic properties of CA1 pyramidal neurons. In hippocampal slices from wild type C57/B6 mice 100 μM PIH application caused an increase in the frequency of spontaneous excitatory postsynaptic currents (EPSCs) (80 ± 14 %, *p* < 0.01, *n* = 16 cells, absolute values depicted in Fig. 2a) without a change in either amplitude (17.5 ± 1.2pA vs 100 μM PIH: 18.8 ± 1.4pA, *p* > 0.3) or decay tau (7.5 ± 0.5 ms vs 100 μM PIH: 7.8 ± 0.5 ms, *p* > 0.3). Decay tau is defined as the time it takes for the signal to return to baseline. In contrast to spontaneous EPSCs, there was no increase in the frequency, amplitude or decay tau of spontaneous IPSCs following PIH treatment (Fig. 2a). Chelating iron also increases the evoked response in a single cell to stimulation of the Schaffer collaterals increasing EPSPs (Fig. 2b, 48 ± 17 %, *p* < 0.02, *n* = 6 cells, individual experiments are depicted below). Next to evaluate differences in the hippocampal circuit response, population EPSPs were induced with a single stimulation to the Schaffer collaterals, and the responses recorded using voltage sensitive dye imaging (VSDi) at 1 kHz. VSDi of hippocampal slices showed an evoked fast depolarization that was followed by a rapid repolarization (Fig. 2c). Application of 100 μM PIH to a hippocampal slice induced an increase in the evoked circuit EPSP response (*n* = 10 slices). Both the amplitude and decay tau were increased following PIH application (Fig. 2c, Amp: 30 ± 4 %, *p* < 0.01; Tau: 75 ± 8 %, *p* < 0.001, *n* = 10, individual experiments are depicted right). When only the vehicle of PIH (5 μM NaOH) was applied to the bath no change in excitability was detected in the slice (data not shown). Moreover, we examined whether PIH treatment affects a ternary complex among Dexras1, ACBD3 and DTM1 as a formation of these complex is essential for Dexras1-mediated iron flux and found that it is not (Fig. 3). Thus, the whole cell and VSDi data show that when intracellular iron is chelated there is an increase in excitability at the single cell and circuit level of the hippocampus.

### NMDA-Rs but not AMPA-Rs are necessary for iron’s effect on evoked EPSP in CA1 of hippocampus

**Fig. 4:**
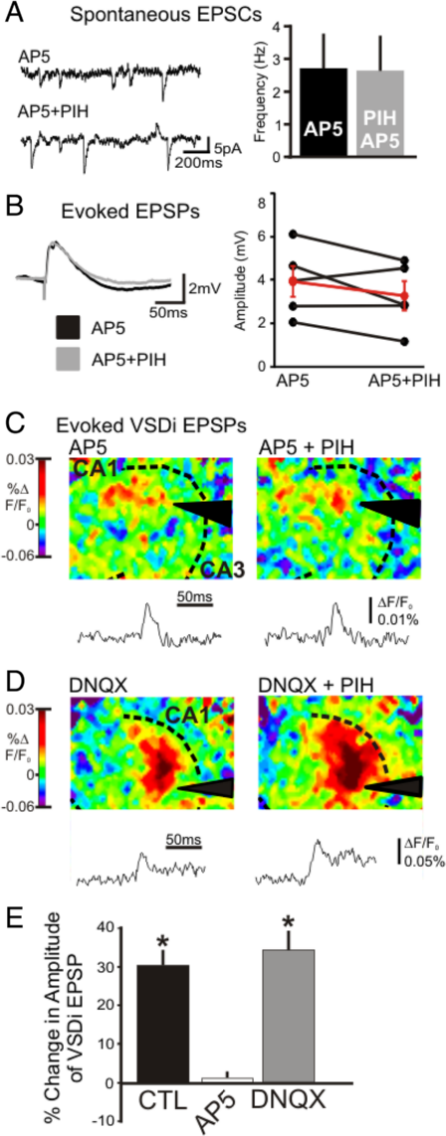
Iron dependent regulation of hippocampal activation is dependent on NMDA not AMPA. **a**. Shows an example of whole cell recordings of WT slices during AP5 and AP5 + 100 μM PIH. Blocking NMDA-R with AP5 abolishes the increase in spontaneous EPSCs. Bar graph shows average of 6 experiments (Control: 2.6 ± 0.9Hz, PIH: 2.5 ± 0.9Hz, *p* = 0.85). **b**. AP-5 blocks or if anything reduces the PIH induced increase in amplitude of the evoked VSDi EPSP (Control: 3.4 ± 0.7 mV, PIH: 2.7 ± 0.7 mV, *n* = 5, *p* = 0.17). **c**. An example of a VSDi experiment with AP5 treatment (*left*) and AP5 with 100 μM PIH added (*right*). The NMDA-R antagonist AP5 blocks the increase in evoked VSDi EPSPs, as shown by the representative traces underneath the snapshots of peak responses. **d**. VSDi analysis of evoked EPSP in the presence of the DNQX (AMPA and kainate receptor antagonist) in low Mg^2+^ ACSF, which are conditions favoring NMDA activity (*left*) and DNQX with PIH on the right. Blocking AMPA and kainate receptors does not have an effect on the VSDi eEPSP. **e**. Blocking NMDA receptors causes PIH to no longer be able to increase the VSDi eEPSP. However only blocking AMPA receptors still allows for an increase in VSDi eEPSP size (*p* < 0.05). WT data is shown for comparison

**Fig. 5:**
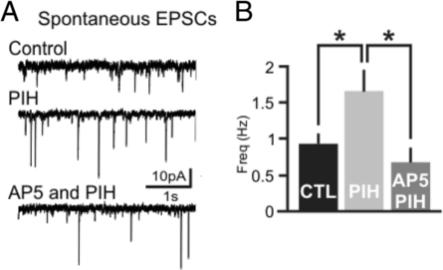
NMDAs R regulate the increase in sEPSC following PIH application. **a**. Adding AP5 to the bath after PIH application causes sEPSC frequency to return to baseline. This shows snap shots of whole cell recordings during initial control (no drug), PIH application, and then AP5 in addition to 100 μM PIH (*n* = 5). **b**. Quantification of sEPSC frequency during PIH and PIH with AP5 normalized to control frequency before any drug application. There is a decrease in EPSC frequency back to baseline after AP5 is added to the bath (*n* = 5 *p < 0.05

Since chelating iron affects the size of the evoked EPSP, we next examined the role of NMDA and AMPA receptors in the iron mediated regulation of hippocampal excitability. Pre-incubation with 50 μM AP5, NMDA-R antagonist, blocked the increase of spontaneous EPSCs induced by iron chelation (Fig. 4a, *n* = 6 cells, absolute values are depicted right). AP5 pre-incubation also blocked the increase in the evoked EPSP (*n* = 5 cells individual cells are depicted in Fig. 4b). NMDA-R receptor activity is also necessary for the population responses examined by VSDi (Fig. 4c, Amp: −0.14 ± 1.8 %, *p* = 0.7; Tau: 1.3 ± 1.4 %, *p* = 0.4, *n* = 5 slices, values of individual experiments are depicted below). When slices are recorded in the presence of AP5, PIH application has no effect on the size of the VSDi eEPSP. In contrast, changes in AMPA receptor activity does not contribute to the iron chelation effect. When DNQX and Lo-Mg^2+^ is present, PIH generates a larger increase in the amplitude and decay tau of the EPSP (Fig. 4d, Amp: 35.2 ± 6 %, *p* < 0.01; Tau: 284.2 ± 26 %, *p* < 0.001, *n* = 5 slices, values of individual experiments are depicted right). When compared to the increased seen when PIH is added to untreated wild type slices, AP5 treated slices did not have an increase in evoked VSDi EPSPs where DNQX treated slices has a similar percentage increase. (*p* < 0.05) (Fig. 4e). Confirming that the increase in excitability was dependent on increased NMDA-R activity we found that applying AP5 after a PIH dependent increase in frequency immediately decreased the EPSC frequency back to baseline (Fig. 5a and b, Control: 0.9 ± 0.13Hz; PIH: 1.6 ± 0.29Hz; AP5 + PIH: 0.66 ± 0.19, *n* = 5 cells).

### Dexras1/DMT1 complex is necessary for the iron dependent modulation of excitability

**Fig. 6:**
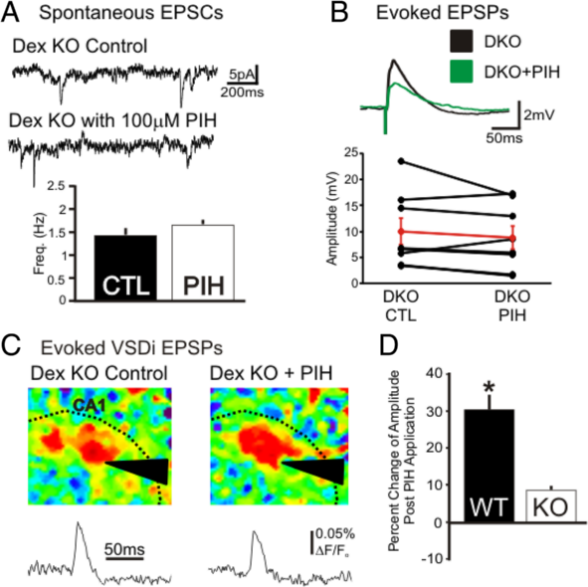
Iron chelation dependent increase in excitability is dependent on Dexras1. **a**. Whole cell patch clamp in Dexras1 KO animals show that there is no effect of chelation on CA1 excitability. PIH has no effect on the frequency of EPSCs in CA1 neurons in Dexras1 KO animals (Control: 1.4 ± 0.3 Hz, PIH: 1.2 ± 0.1 Hz, *n* = 8, *p* = 0.13). PIH application does not increase VSDi eEPSP sized in Dexras KO despite using Low Mg^2+^ ACSF. **b.** Whole cell patch in pyramidal cells of Dexras1 KO also show no increase in evoked EPSPs when PIH is applied On the top is example recordings of whole cell current clamp and bottom is a graph showing population (*black*) and average data (*red)* in the WT animals both before and after 100 μM PIH application (Control: 10.0 ± 2.6 mV, PIH: 8.8 ± 2.3 mV, *n* = 8, *p* = 0.27). **c.** VSDi analysis of Dexras1 KO animals shows that PIH has no effect on the evoked response. An example of a VSDi experiment with Dex KO slices control (*left*) and with 100 μM PIH added (*right*). Directly underneath are average responses averaged over 30 ms post stimulus. **d.** Knocking out Dexras1 causes PIH to no longer have an effect on VSDi eEPSP size

**Fig. 7:**
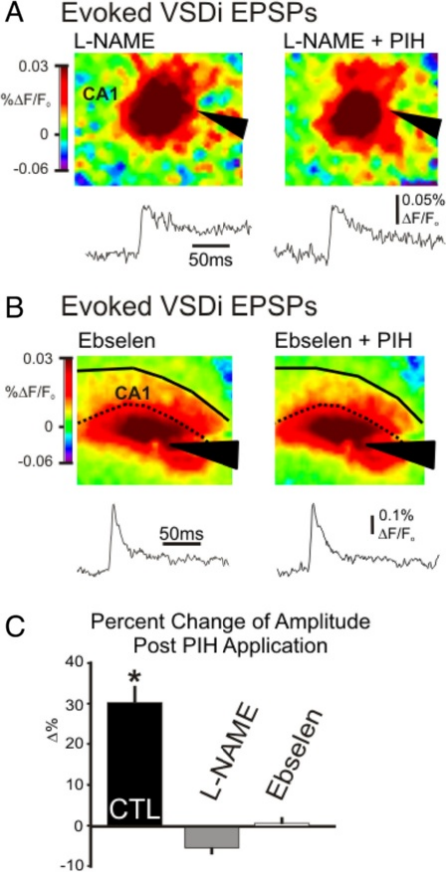
The NO pathway and DMT1 (components of the Dexras1 pathway) are necessary for iron to effect neuronal excitability. **a.** Blocking the NO pathway blocks the PIH induced increase in VSDi eEPEP. An example of a VSDi experiment with L-NAME treatment (*left*) and L-NAME with 100 μM PIH added (*right*). The NO pathway antagonist blocks the increase in evoked VSDi EPSPs, as shown by the representative traces underneath the snapshots of peak responses and population data on the right. (Amp: L-NAME: 1.3x10^−3^ ± 0.1x10^−3^ %ΔF/F_o_, PIH: 1.2x10^−3^ ± 8.3x10^−5^ ΔF/F_o_, *n* = 4, *p* = 0.15). **b.** Blocking DMT1 with ebselen also blocks the PIH induced increase in VSDi eEPEP. An example of a VSDi experiment with ebselen treatment (*left*) and ebselen with 100 μM PIH added (*right*). The DMT1 antagonist blocks the increase in evoked VSDi EPSPs, as shown by the representative traces underneath the snapshots of peak responses and population data on the right (Amp: ebselen: 4.6x10^−3^ ± 0.5x10^−3^ %ΔF/F_o_, PIH: 4.6x10^−3^ ± 0.4x10^−3^ ΔF/F_o_, *n* = 3, *p* = 0.8). **c.** Blocking the NO pathway with L-NAME or blocking DMT1 with ebselen, cause PIH to no longer be able to increase the VSDi eEPSP

In neurons NMDA-mediated activation leads to Dexras1-dependent iron transport via activation of neuronal nitric oxide synthase (nNOS). Following NO dependent *S*-nitrosylation of Dexras1, the protein binds GTP and through its link to a scaffolding protein, ACBD3, increases iron flux through DMT1 [14]. Moreover, our data showed that genetic or pharmacological inhibition of this pathway reduced iron released into the cytosol (Fig. 1c). Hence, we hypothesized that the Dexras1 complex is necessary to generate the cytosolic iron leading to NMDAR-mediated modulation of neuronal excitability. To investigate this role for Dexras1, we performed the same iron chelation experiments on Dexras1 knock out (DexKO) animals. Whole cell patch clamp experiments showed that there were no baseline differences in neuronal excitability as measured by membrane potential, current input–output curve and baseline spontaneous EPSCs. Unlike the wild type mice there was no significant change in sEPSC frequency in DexKO animals when iron is chelated (Fig. 6a, *n* = 8, *p* = 0.13,). Evoked EPSPs were also unaffected in DexKO animals as PIH application had no effect on EPSP amplitude (Fig. 6b, *n* = 6, *p* > 0.05). PIH application in the KO mice caused a much reduced increase on the circuit EPSP when measured by VSDi; an example of this experiment is shown in Fig. 6c (8.4 ± 1.5 % *n* = 4, *p* < 0.02), but a decrease in the decay tau (−15.3 ± 3.3 % *n* = 4, *p* < 0.02,). This increase was a third less than the PIH increase of the evoked circuit EPSP found in WT animals (*p* < 0.01), suggesting that Dexras1 is required for iron dependent NMDAR-mediated modulation of neuronal excitability (Fig. 6d). To directly test signaling through the Dexras1 complex, we performed VSDi experiments while blocking the nitric oxide (NO) pathway as previous studies have shown that NO activation is necessary for Dexras1 to stimulate iron transporter, DMT1 [13, 14]. When the NO pathway was blocked using L-NAME (nitric oxide synthase inhibitor) chelating iron with PIH no longer induced an increase evoked circuit EPSP, if anything a decrease (*n* = 4, *p* = 0.14) however the tau of decay was increased (Fig. 7a, 22.3.4 ± 0.56 %, *p* < 0.01, *n* = 4). We next blocked DMT1 using the small molecular DMT1 transport inhibitor, ebselen, which completely blocked the PIH effect (Fig. 7b, 1.1 ± 2.4 %, *p* = 0.81, *n* = 3). Blocking the proteins associated with the Dexras1 pathway, NO pathway and DMT1, also blocks the increase in VSDi evoked EPSP (Fig. 7c) These data demonstrate that NMDA-R activity is affected by intracellular iron and the same iron-signaling pathway that mediates NMDA neurotoxicity is involved in regulation of cellular excitation, with normal levels of iron likely reducing NMDA-R activity and chelating iron increasing it.

### Iron reduction modulates NMDA-Rs via SRC signaling

**Fig. 8:**
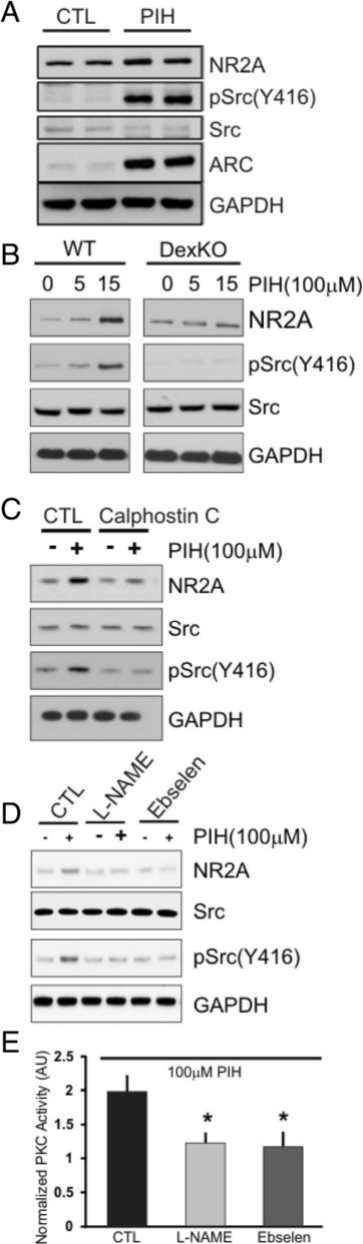
Dexras1-mediated iron flux modulates phosphorylation of NR2A via PKC-Src pathway. **a.** Phosphorylated Src is increased following treatment with PIH. Brain slices from WT mice were treated with 50 μM PIH for 15 min and various proteins were quantified via western blot analysis. ARC, an immediate early gene and important signaling molecule in synaptic plasticity, is elevated as well as pSrc but not unphosphorylated total Src. The bottom band is the internal control GAPDH. **b.** Western blot analysis of brain homogenates show that there are differences in NR2A protein expression between WT and Dexras1 knockout mice and that there is a decrease in the baseline level of phosphorylated pSrc (*n* = 4). PIH application does not induce phosphorylation of Src. The bottom band is the internal control GAPDH. **c.** Hippocampal slices were pre-treated with calphostin C (10 μM) for 15 min and followed by 15 min PIH treatment. Western blot analysis show that calphostin C treatment blocks PIH-mediated activation of pSrc and NR2A. The bottom band is the internal control GAPDH. **d.** Hippocampal slices were pre-treated with either L-NAME (1 mM) or Ebselen (10 uM) for 15 min as described in Fig. 6 and followed by 15 min PIH treatment. Western blot analysis show that these treatments blocks PIH-mediated activation of pSrc and NR2A. The bottom band is the internal control GAPDH. **e.** Iron chelatrion increased PKC activity while inhibition of nNOS or DMT1 blocked PIH-mediated activation of PKC. Brain slices from WT mice were pretreated with either L-NAME or Ebselen and subsequently treated with PIH as described above. Tissues were lysed and PKC activity was measured

Our data showed that chelatable iron pool modulates NMDA-R function and NMDA-R dependent excitability under physiological condition. It has been well documented that NMDA-R function is regulated by Src kinase [27]. Therefore, we first examined whether a 50 μM PIH treatment has any effect on Src signaling. We observed that acute iron chelation in hippocampal slices dramatically increases the levels of phospho-Src (Y416) as well as the levels of NR2A (Fig. 8a). These changes were mediated by the Dexras1 pathway as a deletion of Dexras1 abolished iron chelation-induced phosphorylation of Src (Fig. 8b). Previous data (Fig. 1c) showed that NOS or DMT1 inhibitors blocked iron release into cytosol. Hence we examined whether these agents can reduce pSrc activity. Protein kinase C (PKC) is known to be sensitive to iron levels and is an upstream of Src, through which it also is known to regulate NMDA-R [27]. Treating hippocampal slices with 50 μM PIH in the presence of PKC inhibitor, Calphostin C (10 μM) significantly attenuated PIH-mediated activation of Src and NR2A expression (Fig. 8c). Moreover, we examined whether an inhibitor of nNOS and DMT1 (L-NAME and ebselen, respectively) has any effect on pSrc, NR2A and PKC. We fund that these treatments significantly reduced expression levels of NR2A and pSrc, and activities of PKC (Fig. 8d and e). These experiments demonstrated that the Dexras1 dependent modulation of iron regulates NMDA excitability via PKC/Src signaling.

### The Dexras1 complex is present at the lysosomal membrane

**Fig. 9:**
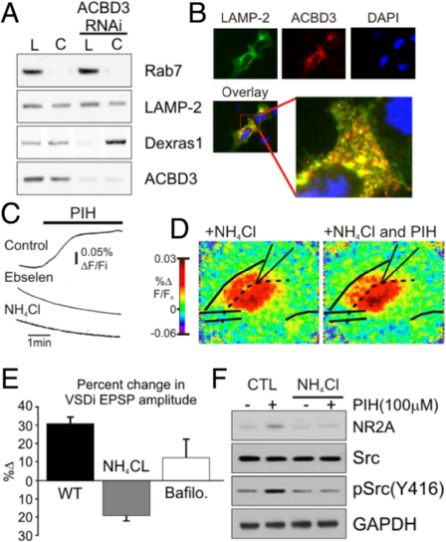
Lysosomes are the source of iron involved with regulating NMDA-R function. **a.** Lysosomal subcellular fraction was isolated and immunoblotted for ACBD3 and Dexras1. Lysosomal specific marker (Rab7) was only detected in the lysosome fraction (L) but not in the cytoplasm fraction (C). **b.** Confocal image shows that ACBD3 is co-localized to lysosome marker LAMP-2. **c.** In Calcein-AM treated slices, PIH is able to de-quench Fe^2+^ from Calcein in CA1, and this effect is blocked by the DMT1 antagonist 10 μM ebselen and as well as 5 mM NH_4_Cl, which collapses the lysosome proton gradient. **d.** VSD imaging of CA1 shows that there is no increase in response to stimulation following PIH application when the lysosome is perturbed with NH_4_Cl. To the right is an averaged response of pixel intensities over time from a standardized region of CA1 in both Control (*black*) and PIH (*red*). **e.** There is no enhancement of the response following PIH application when slices are incubated with NH_4_Cl,(*n* = 6 slices; (*p* = 0.4) of with Bafilomycin, which also perturbs lysosomal release of iron (*n* = 9; *p* = 0.21). **f.** Western blot analysis of brain homogenates before and after PIH treatment when the brains were pre-incubated with NH_4_Cl. Collapsing the lysosomal proton gradient abolishes the phosphorylation of Src

Unlike prior studies of NMDA and iron-dependent neuronal cell death, in these experiments extracellular iron was not added to the extracellular medium nor was a membrane impermeable chelator able to generate the increased neuronal excitability found with PIH indicating that an intracellular source of iron is needed. The lysosome seems well suited to be such a source. A considerable amount of the iron pool can be found in the lysosome [28]. Recent findings from patch-clamping of individual lysosomes show that they can transmit iron through their membrane [29]. Finally, DMT1, which is located on both endo-lysosomal and extracellular membranes, functions more efficiently as a transporter when the pH is low (~5) [30] and the DMT1-isoform 1, which is primarily localized in lysosome, is highly expressed in hippocampus [15, 18]. To examine if the Dexras1 complex with DMT1 is indeed present at the lysosome we isolated lysosomal and cytosolic fractions and tested for Dexras1 and the associated scaffolding protein ACBD3, finding that both Dexras1 and ACBD3 are present in the lysosome. Importantly, ACBD3 knockdown by RNAi significantly reduced a level of Dexras1 associated with a lysosomal fraction (Fig. 9a) suggesting that Dexras1 is localized in lysosome via ACBD3. Additionally, confocal imaging analysis revealed that ACBD3 is highly co-localized with LAMP-2, a lysosomal marker, corroborating that a ternary complex of Dexras1/ACBD3/DMT1 can be formed on the lysosome (Fig. 9b).

After establishing that the elements of the Dexras1/DMT1 complex are at the lysosome, we investigated whether the lysosome is involved in the iron signaling. We disrupted lysosomal function using 5 mM NH_4_Cl, to compromise the pH gradient and examined the presence of cheatable iron in CA1, the ability of PIH to increase excitability and Src phosphorylation. As shown in Fig. 9c we found that collapsing the proton gradient reduced the PIH-induced change in fluorescence in calcein-AM dye signal as similar to the treatment with ebselen1, which blocks DMT1 channel. In the VSDi experiments NMDA EPSPs were isolated pharmacologically limiting the impact of NH_4_Cl to NMDA excitability. Because 5 mM NH_4_Cl can impact hippocampal excitability itself, it is important that under these conditions, baseline responses in ACSF containing NH_4_Cl did not appear different from untreated slices. Under these conditions PIH did not increase excitability (Fig. 9d, −19.3.4 ± 2.7 %, *p* = 0.21, *n* = 6 slices, individual experiments are graphed right). We further confirmed that bafilomycin, an inhibitor of lysosomal function also reduced PIH-mediated ESPS (Fig. 9e; NH_4_Cl: −19 ± 2.7 % *n* = 6, *p* > 0.2; 1 μM Bafiliomycin: 12 ± 10 % *n* = 8, *p* > 0.2). Slices treated with NH_4_Cl also lost iron chelator-mediated activation of Src (Fig. 9f). These data are consistent with the hypothesis that an intact lysosome is required for the intracellular iron flux modulating NMDA dependent neuronal excitability.

## Disscusion

In this paper we have demonstrated that intracellular iron signaling can modulate neuronal excitability in the hippocampus. We show that iron is released in neurons when stimulated either by KCl or via synaptic stimulation and in turn, this release can regulate neuronal excitability. When intracellular iron is reduced, Schaffer evoked synaptic responses in CA1 as well as spontaneous EPSCs were increased. NMDA-R and kinase pathways controlling NMDA-R activity were necessary for this rapid increase in excitability. By extension it is likely that iron acts to suppress NMDA-R dependent excitability in response to excitation. The small GTPase Dexras1 and its associated partners including the metal transporter DMT1 were also necessary for the iron-mediated regulation of excitability. In contrast to conditions leading to cell death, physiological levels of iron appear to suppress NMDA dependent changes in excitability and thus iron at physiological levels may limit the potential for excitotoxicity.

**Fig. 10:**
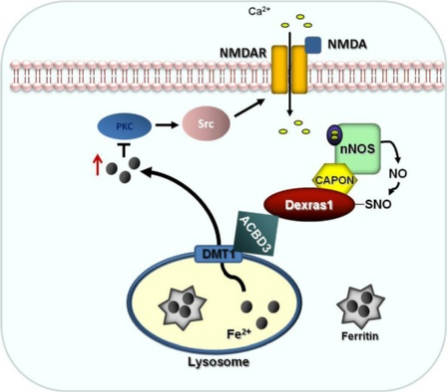
Iron Modulates NMDA-R function via Src and PKC pathway which then mediates neuronal excitability. NMDA-mediated activation of Dexras1 modulates iron release from intracellular source, lysosome via iron transporter, DMT1 and this consequently suppresses synaptic NMDA activity by suppressing PKC/Src pathway. Therefore, Dexras1-mediated iron modulation in neurons can act in a feedback manner to modulate NMDA function and thus may exert homeostatic control of excitability

Lack of extracellular iron or cell impermeable chelators had no impact on excitability, indicating that intracellular release is sufficient to dampen these changes in excitability. Our studies point to the lysosome as the most likely source of the intracellular iron flux. The Dexras1 complex is present at the lysosome and when the lysosomal proton gradient is collapsed chelatable iron is reduced and the chelation-dependent increase in excitability is lost. Together these data chart a pathway where intracellular iron released from intracellular stores regulates neuronal excitability by decreasing NMDA receptor mediated excitation of pyramidal neurons in the hippocampus (Fig. 10).

### NMDA-R dependent increase in spontaneous EPSCs

One of the most robust findings was an iron chelation dependent increase in spontaneous EPSCs frequency. At first we saw this increase in frequency as likely due to a separate non-NMDA-dependent presynaptic effect of iron chelation. However, the ability of the NMDA-R antagonist AP5 to block, as well as reverse the PIH dependent increases in frequency again demonstrated a central role for NMDA-R. The best explanation is reflected in a presynaptic NMDA-R role in modulating spontaneous release found through recordings of miniature (action potential independent) EPSCs or spontaneous EPSCs [31, 32]. In Kunz et al. disrupting intracellular calcium signaling increased frequency, but not amplitude of mEPSCs, as in our work this frequency increase could be reversed by AP5 [31]. Thus, while our studies target different intracellular messenger systems, our results converge on presynaptic NMDA-Rs to modulate spontaneous synaptic release.

### Sources of iron in the neuron

Iron is abundant in the brain [33] and yet most of the studies of iron functionality in the brain have centered on its pathophysiological properties as a catalyst in the production of ROS that contribute to neurodegeneration [34]. A more physiological role of iron is known to regulate the activities of enzymes that control the synthesis of neurotransmitters, and in particular catecholamines [33, 35]. A lack of iron leads to aberrant neuronal activities, such as impairment in hippocampal electrophysiology, disrupted CA1 apical dendrite structure, and deficits in learning and memory [10, 11]. These observations suggest that iron in the brain may play a role in signaling. Physiologically, the majority of cells in the body acquire iron from a well-characterized plasma glycoprotein by receptor-mediated endocytosis through the transferrin receptor. Any excess iron delivered to the cytoplasm is transported to a storage protein, ferritin [16, 36–38], which is recycled by lysosomes [28, 39]. Previous literature suggested that iron in ferritin can be released, but this process is extremely slow because iron is in a mineralized form. Thus, ferritin bound iron likely has little contribution to the intracellular iron described in the results [40, 41]**.** However, that ferritin is degraded in the lysosome [38, 39, 42]. When this happens the iron is released where iron’s solubility is aided by the acidic environment of the lysosome. While there are very few studies investigating the mechanism by which lysosomal iron is recycled, it has been reported that iron can be also released from lysosome via type IV mucolipidosis-associated protein TRPML1 [29]. Therefore, our data and current literature strongly support the notion that lysosomal iron may be a source for a bio-active iron, which can be immediately utilized for physiological functions.

### Dexras1 complex at the lysosome

Interestingly, DMT1-isoform I, which is primarily targeted to the lysosome, is highly expressed in the hippocampus, where we observed a significant presence of intracellular iron. We also determined that the Dexras1/ACBD3/DMT1 ternary complex appears present on the lysosome. Thus, our current studies further extend the role of Dexras1 as a modulator of intracellular iron and broaden the possible roles of this intracellular source of iron. In fact, DMT1 may be better able to play a role in ferritin iron recycling from lysosome [39, 41], since iron flux activity of DMT1 is several folds higher at low pH.

### Potentially confounding metals

It is prudent to mention that DMT1 can transport other various metals such as Cu, Zn or Mn and as in any similar studies our dyes and chelators can also bind other metals, generating potential confounds. Moreover, it has been shown that Cu as well as Mn can inhibit NMDA–R function. Yet, the Ki values of these metals are above 100 μM, which are levels usually only reached under pathological or experimental conditions [43]. We have further excluded a possibility of Cu, as the Cu-chelator, tetrathiomolybdate, did not mimic the effect of iron chelation. Zn has also been extensively studied in neurological system, impacts NMDA-R activity and is involved in the most established metal signaling mechanisms in the brain [44–49]. We can speculate that Zn-released from lysosome may explain our observations as DMT1 can transport Zn. However, it is unlikely that Zn is playing a role in our data. First, the chelator we utilized, PIH, is selective for iron over Zn [22]. Secondly, Zn containing neurons are extensively concentrated in the dentate gyrus and mossy fibers that innervate CA3 [50] and our imaging data detected the greatest concentration of cheatable metal in hippocampal area CA1. Moreover, Zn is known to predominantly inhibit NMDA-R function by directly binding to the receptor at an extracellular domain [51] and the increased in NMDA-R dependent EPSPs was only generated by membrane permeable chelators. Because vesicular Zn is present in area CA1 and intracellular Zn can act on the post-synaptic scaffolding proteins, a role of Zn cannot completely be ruled out, though intracellular Zn acting on PKC has not been reported [52]. Thus, intracellular iron released from lysosome remains the most likely candidate for intracellular metal dependent modulation of NMDA-R function, highlighting a novel form of metal signaling within the cell.

### Role for Dexras1 complex and iron on NMDA-R activity

This work finds that reducing iron can enhance NMDA-R excitability, via PKC and Src dependent signaling. This is consistent with prior work that iron chelation can activate the PKC pathway in other tissue [53, 54]. As recent literature suggests this change in PKC activity may be through ROS associated with iron, which can be potent signaling molecules [55–57]. ROS generation can indeed impact NMDA-R function and our data may elucidate a molecular signaling pathway in which NMDA-mediated re-distribution of iron can precisely control the NMDA-R function through ROS generation. Together, the prior work demonstrating a Dexras1 and NMDA dependent increase in iron, and the present study illustrating how Dexras1 dependent iron release my suppress NMDA activity, indicates that Dexras1 signaling and iron can act in a feedback manner to modulate NMDA function and thus may exert homeostatic control of excitability. Additionally, the potential feedback role of this mechanism may explain the phenomena of NMDA dependent preconditioning where low levels of NMDA-R activity can reduce later NMDA dependent excitotoxic damage in models of neurodegeneration [13, 14].

In identifying a unique target for modulating NMDA-R, we in turn potentially target a host of neuronal functions dependent on NMDA-Rs. In fact, this pathway may suggest a novel mechanism of action for the DMT1 channel blocker, ebselen, which has shown promise as a replacement for psychiatric uses of lithium [58]. The physiological significance of iron acting on NMDA-R activity is heightened by robust affects on both pre and post-synaptic excitability. Because NMDA-R activity in area CA1 and other parts of the brain mediates homeostasis, development and plasticity central to learning and memory mechanisms, iron modulation of NMDA-R activity through the Dexras1 dependent pathway may be an important component of these processes. The current establishment of this molecular pathway and the impact of iron on NMDA dependent synaptic excitability sets the stage for targeting iron and the Dexras1 pathways in learning and other behaviors.

## Conclusions

We found that genetic and pharmacological ablation of this pathway increased glutamatergic transmission. Voltage sensitive dye imaging of hippocampal slices and whole-cell patch clamping of synaptic currents, indicated that the increase in excitability was due to synaptic modification of NMDA-R activity via modulation of the PKC/Src/NR2A pathway. Moreover, we identified that lysosomal iron serves as a main source for intracellular iron signaling modulating NMDA-R mediated glutamatergic excitability. These data reveal a unique intracellular role of iron to robustly modulate synaptic excitability under physiological conditions of iron in neurons.

## Methods

### Animals

All animals (wild type “male” C57BL/6 and Dexras1/RASD1 KO) were housed 4–5 per cage on a 12-h light/dark cycle in a temperature-controlled facility with food and water available ad libitum. All experiments were performed during their light cycle when they were 2–6 month of age. All protocols for their care and testing were performed in accordance with University Laboratory Animal Resources guidelines and approved by the Institutional animal Care and Use Committee. Dexras1/RASD1 knock out animals were genotyped with PCR as explained in [13].

### Pharmacological agents

50*–*100 μM pyridoxal isonicotinoyl hydrazone (PIH), a membrane permeable iron chelator, dissolved in 5 mM NaOH (final concentration 5 μM) was used in most of the following experiments to chelate iron. 50 μM (2R)-amino-5-phosphonopentanoate (AP5), was used to block NMDA-R and 50 μM 6, 7-dinitroquinoxaline-2, 3-dione (DNQX), was used to block AMPA and Kainate receptors. 10 μM ebselen was used to block divalent metal transporter 1 (DMT1). One mM L-NAME was used to block the nitrous oxide synthase pathway. To collapse the lysosomal proton gradient 5 mM NH_4_Cl was applied in the ACSF.

### Single cell iron imaging

Primary hippocampal neurons were prepared as described before [14, 59]. Cells were washed with warm PBS (×2) and incubated with 20 μM Calcien-AM (Inivtrogen) for 30 min in PBS. Cells were washed with Kreb’s buffer (118 mM NaCl, 4.7 mM KCl, 1.2 mM KH_2_PO_4_, 1.2 mM MgSO_4_, 4.2 mM NaHCO_3_, 2 mM CaCl_2_, 10 mM glucose, 200 mM sulphinpyrazone and 10 mM Hepes, pH 7.4) twice. Cells were placed in 1 mM Kreb’s buffer containing 50 mM KCl and immunofluorescence was measured by LEICA TCS SP5 confocal microscope (excitation 495 nm, emission 515 nm). Five to ten minutes later, 100 μM of PIH was added into buffer and fluorescence signal was measured.

### Transverse hippocampal slice iron imaging

Mice between 3 to 4 weeks-old were decapitated following brief isoflurane anesthesia. The brain was removed and transverse slices (350 μm) containing the hippocampus were cut with a vibratome in an ice-cold artificial cerebrospinal fluid solution (ACSF), in which NaCl was replaced by an equiosmolar concentration of sucrose. ACSF consisted of 130 mM NaCl, 3 mM KCl, 1.25 mM NaH_2_PO_4_, 26 mM NaHCO_3_, 10 mM glucose, 1 mM MgCl_2_, and 2 mM CaCl_2_ (pH 7.2–7.4 when saturated with 95 % O_2_/5 % CO_2_). Slices were incubated in ACSF at 32–34 °C for 30 min and kept at 22–25 °C thereafter, until transfer to the recording chamber. The osmolarity of all extracellular solutions was 305–315 mOsm. Slices were viewed using infrared differential interference contrast optics under an upright microscope (Eclipse FN1, Nikon Instruments) with a 40 × water-immersion objective.

### Electrophysiology

#### Voltage sensitive dye imaging

Two month old wild type and dexras1−/− (DexKO) mice were decapitated following isoflurane anesthesia. The brain was removed and horizontal hippocampal slices (350 μm) were cut with an Integraslice 7550 PSDS (Campden Instruments, Lafayette, IN) in an ice-cold artificial cerebrospinal fluid (ACSF) containing an equiosmolar concentration of sucrose. Recording Na-ACSF consisted of: 130 mM NaCl, 3 KCl, 1.25 mM NaH_2_PO_4_, 26 mM NaHCO_3_, 10 mM glucose, 1 mM MgCl_2_, 2 mM CaCl_2_ (pH 7.2–7.4 when saturated with 95 % O_2_/5 % CO_2_). Hippocampal slices (between −5.60 and −6.60 from Bregma) were then transferred to a static interface chamber (34 °C) for 30 min and kept at 22–25 °C thereafter, until transfer to the recording chamber. The osmolarity of all solutions was 305–315 mOsm. To focus on NMDA dependent synaptic activity, many experiments (DexKO and NH_4_Cl VSDi conditions) were done with a Lo-Mg^2+^ ACSF, which consisted of: 130 mM NaCl, 3 mM KCl, 1.1 mM NaH_2_PO_4_, 26 mM NaHCO_3_, 10 mM glucose, 0.1 mM MgCl_2_, and 2 mM CaCl_2_ (pH 7.2–7.4 when saturated with 95 % O_2_/5 % CO_2_) in the presence of DNQX.

Slices were stained with 0.125 mg/ml of the voltage-sensitive dye di-3-ANEPPDHQ (D36801, Invitrogen) in ACSF for 20 min, and imaged in an oxygenated interface chamber (34 °C) using an 80 × 80 CCD camera recording at a 2-kHz frame rate (NeuroCCD: RedShirtImaging, Decatur, GA). Epi-illumination was provided by a custom LED illuminator. Compared with the more commonly used photodiode array, the CCD chip well size (215,000 electrons) requires use of relatively low light intensities minimizing photodynamic damage. A 10 × objective lens (Olympus, Tokyo, Japan) imaged a 2.5 × 2.5 mm CA1 region (32 × 32 μm region per pixel). Schaffer collateral stimulation was administered with the electrode placed near the border of CA1 in the stratum radiatum (SR). Each trial consisted of 12 stimuli, 20 μs apart.

### Whole-cell patch recording

A recording chamber for submerged brain slices was continuously perfused (1–2 ml/min) with oxygenated ACSF heated to 32 ± 1 °C using an automatic temperature controller (Warner Instruments). Recording pipettes were pulled from borosilicate glass capillaries (World Precision Instruments) to a resistance of 3–7 MΩ when filled with the intracellular solution. For voltage clamp and spontaneous PSC recordings, the intracellular solution contained: 145 mM potassium gluconate, 2 mM MgCl_2_, 2.5 mM KCl, 2.5 mM NaCl, 0.1 mM BAPTA, 10 mM HEPES, 2 mM Mg-ATP, 0.5 mM GTP-Tris, and 5 mM QX-314 (pH 7.2–7.3 with KOH, osmolarity 280–290 mOsm). Evoked PSCs and Spontaneous PSC recordings were conducted in whole-cell voltage-clamp mode (V_h_ =–70 mV for spontaneous EPSCs and V_h_ = 0 mV for spontaneous IPSCs). All recordings were conducted with a MultiClamp700B amplifier (Molecular Devices). Currents were low-pass filtered at 2 kHz and digitized at 20 kHz using a Digidata 1440A acquisition board and pClamp10 software (both from Molecular Devices). Access resistance (10–30 MΩ) was monitored throughout the recordings by injection of 10 mV hyperpolarizing pulses and data were discarded if access resistance changed by >25 % over the course of data acquisition. Evoked responses were triggered by 100 μs constant-current pulses generated by an A310 Accupulser (World Precision Instruments) and delivered at 0.1 Hz via a bipolar tungsten stimulation electrode positioned within 100 μm of the recorded cell. The amplitude of the current pulses was controlled by a stimulus isolator (ISO-Flex, AMPI) and was adjusted to elicit monosynaptic responses in the range of 50–300 pA.

### Ex vivo stimulation and immunoblotting

Hippocampi from mice were cut at 350 μM intervals in sagittal and coronal planes using a McIlwain tissue chopper. The slices were dispersed in oxygenated artificial cerebrospinal fluid buffer (ACSF, 113 mM NaCl, 4.5 mM KCl, 1 mM MgCl_2_, 25 mM NaHCO_3_, 1 mM NaH_2_PO_4_, 25 mM Glucose, 2 mM CaCl_2_) and then incubated with reagents (Sigma, St. Louis, MO) for 15 min and washed with ice cold ACSF [60, 61]. Tissues were lysed in lysis buffer and subjected to SDS-PAGE and immunoblot, as previously described [13, 61, 62–64] Antibodies for NR2A, pSRc (Y416), Src with GAPDH as a loading control were from Cell Signaling, (Danvers, MA) and Arc antibody is from Santa Cruz Biotechnology (Santa Cruz, CA).

### Cellular fractionation

Lysosome fractions were isolated by lysosome enrichment kit for tissue and cell culture from Thermo Scientific according to manufacturer’s instruction.

### Immunofluorescence

PC-12 cells were seeded on glass cover-slips as described before [62]. LAMP2 and ACBD3 antibody were purchased from Abcam (Eugene, OR). Bound antibodies were visualized using Alexa Fluor 594 goat anti-mouse IgGs and 488 goat anti-rabbit IgGs (Invitrogen). Confocal microscopy analysis was performed under oil immersion on Leica DMI6000 (Leica Microsystems) with a 63× objective [62].

## Abbreviation

ACBD3: acyl coa binding domain 3
AMPA: α-amino-3-hydroxy-5-methyl-4-isoxazolepropionic acid
DexKO: Dexras1 knock out
DMTA: divalent metal transporter 1
EPSC: excitatory postsynaptic currents
IPSC: inhibitory postsynaptic currents
NMDA: N-methyl-D-aspartate, NMDA
nNOS: neuronal nitric oxide synthase
NO: nitric oxide
NR: NMDA receptor
PC12: adrenal pheochromocytoma
PIH: pyridoxal isonicotinoyl hydrazine
PKC: protein kinase C
VSDi: voltage sensitive dye imaging

## References

[1] Rötig A, Sidi D, Munnich A, Rustin P (2002). Molecular insights into Friedreich’s ataxia and antioxidant-based therapies. Trends Mol Med.

[2] Pandolfo M (2002). Iron Metabolism and Mitochondrial Abnormalities in Friedreich Ataxia. Blood Cells Mol Dis.

[3] Koeppen A, Dickson A (2001). Iron in the Hallervorden-Spatz syndrome. Pediatr Neurol.

[4] Gitlin JD (1998). Aceruloplasminemia(Review). Pediatr Res.

[5] Berg D, Gerlach M, Youdim MBH, Double KL, Zecca L, Riederer P, Becker G: Brain iron pathways and their relevance to Parkinson’s disease. J Neurochem. 2008;79:225–36. 10.1046/j.1471-4159.2001.00608.x 11677250

[6] Carlson ES, Stead JDH, Neal CR, Petryk A, Georgieff MK (2007). Perinatal iron deficiency results in altered developmental expression of genes mediating energy metabolism and neuronal morphogenesis in hippocampus. Hippocampus.

[7] McGrath J, Brown A, St Clair D (2011). Prevention and schizophrenia--the role of dietary factors. Schizophr Bull.

[8] Sidrak S, Yoong T, Woolfenden S (2014). Iron deficiency in children with global developmental delay and autism spectrum disorder. J Paediatr Child Health.

[9] Chen M-H, Su T-P, Chen Y-S, Hsu J-W, Huang K-L, Chang W-H, Chen T-J, Bai Y-M: Association between psychiatric disorders and iron deficiency anemia among children and adolescents: a nationwide population-based study. BMC Psychiatry. 2013;13:161. 10.1186/1471-244X-13-161 PMC3680022 23735056

[10] Fretham SJB, Carlson ES, Georgieff MK (2011). The Role of Iron in Learning and Memory. Adv Nutr.

[11] Muñoz P, Humeres A (2012). Iron deficiency on neuronal function. Biometals.

[12] Muñoz P, Humeres A, Elgueta C, Kirkwood A, Hidalgo C, Núñez MT (2011). Iron mediates N-methyl-D-aspartate receptor-dependent stimulation of calcium-induced pathways and hippocampal synaptic plasticity. J Biol Chem.

[13] Chen Y, Khan RS, Cwanger A, Song Y, Steenstra C, Bang S, Cheah JH, Dunaief J, Shindler KS, Snyder SH, Kim SF (2013). Dexras1, a small GTPase, is required for glutamate-NMDA neurotoxicity. J Neurosci.

[14] Cheah JH, Kim SF, Hester LD, Clancy KW, Patterson SE, Papadopoulos V, Snyder SH: NMDA receptor-nitric oxide transmission mediates neuronal iron homeostasis via the GTPase Dexras1. Neuron. 2006;51:431–40. 10.1016/j.neuron.2006.07.011 PMC3150500 16908409

[15] Pelizzoni I, Zacchetti D, Smith CP, Grohovaz F, Codazzi F (2012). Expression of divalent metal transporter 1 in primary hippocampal neurons: reconsidering its role in non-transferrin-bound iron influx. J Neurochem.

[16] Muckenthaler MU, Galy B, Hentze MW (2008). Systemic iron homeostasis and the iron-responsive element/iron-regulatory protein (IRE/IRP) regulatory network. Annu Rev Nutr.

[17] Garrick MD (2011). Human iron transporters. Genes Nutr.

[18] Lam-Yuk-Tseung S, Gros P (2006). Distinct Targeting and Recycling Properties of Two Isoforms of the Iron. Biochemistry.

[19] Mackenzie B, Takanaga H, Hubert N, Rolfs A, Hediger M a (2007). Functional properties of multiple isoforms of human divalent metal-ion transporter 1 (DMT1). Biochem J.

[20] Epsztejn S, Kakhlon O, Glickstein H, Breuer W, Cabantchik I (1997). Fluorescence analysis of the labile iron pool of mammalian cells. Anal Biochem.

[21] Thomas F, Serratrice G, Béguin C, Aman ES, Pierre JL, Fontecave M, Laulhere JP: Calcein as a fluorescent probe for ferric iron. J Biol Chem. 1999;274:13375–83. 10.1074/jbc.274.19.13375 10224100

[22] Richardson D, Hefter G, May P, Webb J, Baker E (1989). Iron chelators of the pyridoxal isonicotinoyl hydrazone class III: Formation constants with calcium(II), magnesium(II) and zinc(II). Biol Met.

[23] Breuer W, Epsztejn S, Cabantchik ZI (1995). Iron acquired from transferrin by K562 cells is delivered into a cytoplasmic pool of chelatable iron(II). J Biol Chem.

[24] Kakhlon O, Cabantchik ZI (2002). Introduction-serial review: iron and cellular redox status. Free Radic Biol Med.

[25] Petrat F, Rauen U, de Groot H (1999). Determination of the chelatable iron pool of isolated rat hepatocytes by digital fluorescence microscopy using the fluorescent probe, phen green SK. Hepatology.

[26] Petrat F, de Groot H, Rauen U (2001). Subcellular distribution of chelatable iron : a laser scanning microscopic study in isolated hepatocytes and liver endothelial cells. Biochemistry.

[27] Lu WY, Xiong ZG, Lei S, Orser B a, Dudek E, Browning MD, MacDonald JF (1999). G-protein-coupled receptors act via protein kinase C and Src to regulate NMDA receptors. Nat Neurosci.

[28] Kurz T, Eaton JW, Brunk UT (2011). The role of lysosomes in iron metabolism and recycling. Int J Biochem Cell Biol.

[29] Dong X-P, Cheng X, Mills E, Delling M, Wang F, Kurz T, Xu H (2008). The type IV mucolipidosis-associated protein TRPML1 is an endolysosomal iron release channel. Nature.

[30] Mackenzie B, Ujwal ML, Chang M-H, Romero MF, Hediger M a (2006). Divalent metal-ion transporter DMT1 mediates both H+ −coupled Fe2+ transport and uncoupled fluxes. Pflugers Arch.

[31] Kunz P a, Roberts AC, Philpot BD (2013). Presynaptic NMDA receptor mechanisms for enhancing spontaneous neurotransmitter release. J Neurosci.

[32] Berretta N, Jones R (1996). Tonic facilitation of glutamate release by presynaptic N-methyl-d-aspartate autoreceptors in the entorhinal cortex. Neurosci Lett.

[33] Crichton RR, Dexter DT, Ward RJ (2011). Brain iron metabolism and its perturbation in neurological diseases. J Neural Transm.

[34] Snyder AM, Connor JR (2009). Iron, the substantia nigra and related neurological disorders. Biochim Biophys Acta.

[35] Zecca L, Youdim MBH, Riederer P, Connor JR, Crichton RR (2004). Iron, brain ageing and neurodegenerative disorders. Nat Rev Neurosci.

[36] Andrews NC (2008). Forging a field: the golden age of iron biology. Blood.

[37] Kim S, Ponka P (2000). Effects of Interferon- γ and Lipopolysaccharide on Macrophage Iron Metabolism Are Mediated by Nitric Oxide-induced Degradation of Iron Regulatory Protein 2. J Biol Chem.

[38] Kim S, Ponka P (2002). Nitrogen monoxide-mediated control of ferritin synthesis: Implications for macrophage iron homeostasis. Proc Natl Acad Sci U S A.

[39] Kidane TZ, Sauble E, Linder MC (2006). Release of iron from ferritin requires lysosomal activity. Am J Physiol Cell Physiol.

[40] Theil EC, Matzapetakis M, Liu X (2006). Ferritins : iron/oxygen biominerals in protein nanocages. J Biol Inorg Chem.

[41] Arosio P, Ingrassia R, Cavadini P (2009). Ferritins: A family of molecules for iron storage, antioxidation and more. Biochim Biophys Acta.

[42] Asano T, Komatsu M, Yamaguchi-Iwai Y, Ishikawa F, Mizushima N, Iwai K (2011). Distinct mechanisms of ferritin delivery to lysosomes in iron-depleted and iron-replete cells. Mol Cell Biol.

[43] Guilarte TR, Chen M-K (2007). Manganese inhibits NMDA receptor channel function: implications to psychiatric and cognitive effects. Neurotoxicology.

[44] Vergnano AM, Rebola N, Savtchenko LP, Pinheiro PS, Casado M, Kieffer BL, Rusakov DA, Mulle C, Paoletti P (2014). Zinc Dynamics and Action at Excitatory Synapses. Neuron.

[45] Cuajungco MP, Lees GJ (1997). Zinc metabolism in the brain: relevance to human neurodegenerative disorders. Neurobiol Dis.

[46] McCord MC, Aizenman E (2014). The role of intracellular zinc release in aging, oxidative stress, and Alzheimer’s disease. Front Aging Neurosci.

[47] Lau A, Tymianski M (2010). Glutamate receptors, neurotoxicity and neurodegeneration. Pflugers Arch.

[48] Smart TG, Hosie AM, Miller PS (2004). Zn2+ ions: modulators of excitatory and inhibitory synaptic activity. Neuroscientist.

[49] Takeda A, Nakamura M, Fujii H, Tamano H (2013). Synaptic Zn(2+) homeostasis and its significance. Metallomics.

[50] Frederickson RE, Frederickson CJ, Danscher G (1990). In situ binding of bouton zinc reversibly disrupts performance on a spatial memory task. Behav Brain Res.

[51] Paoletti P (2011). Molecular basis of NMDA receptor functional diversity. Eur J Neurosci.

[52] Sindreu C, Bayés Á, Altafaj X, Pérez-Clausell J (2014). Zinc transporter-1 concentrates at the postsynaptic density of hippocampal synapses. Mol Brain.

[53] Choi E-Y, Lee S, Oh H-M, Kim Y-D, Choi E-J, Kim S-H, Kim S-W, Choi S-C, Jun C-D (2007). Involvement of protein kinase Cdelta in iron chelator-induced IL-8 production in human intestinal epithelial cells. Life Sci.

[54] Wiesinger J a, Buwen JP, Buwen CJ, Unger EL, Jones BC, Beard JL (2007). Down-regulation of dopamine transporter by iron chelation in vitro is mediated by altered trafficking, not synthesis. J Neurochem.

[55] Murphy MP, Holmgren A, Larsson N-G, Halliwell B, Chang CJ, Kalyanaraman B, Rhee SG, Thornalley PJ, Partridge L, Gems D, Nyström T, Belousov V, Schumacker PT, Winterbourn CC (2011). Unraveling the biological roles of reactive oxygen species. Cell Metab.

[56] Bae YS, Oh H, Rhee SG, Yoo YD (2011). Regulation of reactive oxygen species generation in cell signaling. Mol Cells.

[57] Holmström KM, Finkel T (2014). Cellular mechanisms and physiological consequences of redox-dependent signalling. Nat Rev Mol Cell Biol.

[58] Singh N, Halliday AC, Thomas JM, Kuznetsova OV, Baldwin R, Woon ECY, Aley PK, Antoniadou I, Sharp T, Vasudevan SR, Churchill GC (2013). A safe lithium mimetic for bipolar disorder. Nat Commun.

[59] Tian J, Kim SF, Hester L, Snyder SH (2008). S-nitrosylation/activation of COX-2 mediates NMDA neurotoxicity. Proc Natl Acad Sci U S A..

[60] Kim SF, Huang AS, Snowman AM, Teuscher C, Snyder SH (2007). Antipsychotic drug-induced weight gain mediated by histamine H1 receptor-linked activation of hypothalamic AMP-kinase. Proc Natl Acad Sci U S A..

[61] Arnold SE, Lucki I, Brookshire BR, Carlson GC (2014). Browne C a, Kazi H, Snyder SH: High fat diet produces brain insulin resistance, synaptodendritic abnormalities and altered behavior in mice. Neurobiol Dis..

[62] Bang S, Steenstra C, Kim SF (2012). Striatum specific protein, Rhes regulates AKT pathway. Neurosci Lett..

[63] Kim S, Kim SF, Maag D, Maxwell MJ, Resnick AC, Juluri KR, Chakraborty A (2011). Koldobskiy M a, Cha SH, Barrow R, Snowman AM, Snyder SH: Amino acid signaling to mTOR mediated by inositol polyphosphate multikinase. Cell Metab..

[64] Chakraborty A (2010). Koldobskiy M a, Bello NT, Maxwell M, Potter JJ, Juluri KR, Maag D, Kim S, Huang AS, Dailey MJ, Saleh M, Snowman AM, Moran TH, Mezey E, Snyder SH. Inositol pyrophosphates inhibit Akt signaling, thereby regulating insulin sensitivity and weight gain. Cell.

